# Clinical features of multiple primary carcinomas of the oral cavity

**DOI:** 10.3892/etm.2019.7832

**Published:** 2019-07-30

**Authors:** Ya-Dong Li, Xin Ma, Yao-Lun Han, Li-Wei Peng

Exp Ther Med 13: 634-638, 2017; DOI: 10.3892/etm.2016.3999

An interested reader drew to our attention the fact that quite a subtantial amount of the text, as well as Figs. 1–3, that appeared in the above article had already appeared in the following paper [Mochizuki Y, Harada H, Ikuta M, Shimamoto H, Tomioka H, Tanaka K, Hirai H and Omura K: Clinical characteristics of multiple primary carcinomas of the oral cavity. Oral Oncol 52: 182–189, 2015].

Following an investigation, the Journal was able to confirm that this accusation of plagiarism is well-founded. On those grounds, the Editor of *Experimental and Theraputic Medicine* has decided to retract this paper. Every effort was made to contact the authors in this regard, but without success. The Editor deeply regrets any inconvenience that this retraction has caused to the readership of the Journal or to the authors of the previously published paper.

